# Maternally-derived antibodies do not prevent transmission of swine influenza A virus between pigs

**DOI:** 10.1186/s13567-016-0365-6

**Published:** 2016-08-17

**Authors:** Charlie Cador, Séverine Hervé, Mathieu Andraud, Stéphane Gorin, Frédéric Paboeuf, Nicolas Barbier, Stéphane Quéguiner, Céline Deblanc, Gaëlle Simon, Nicolas Rose

**Affiliations:** 1 Swine Epidemiology and Welfare Research Unit, French Agency for Food, Environmental and Occupational Health & Safety (ANSES), BP 53, 22440 Ploufragan, France; 2 Swine Virology Immunology Research Unit, French Agency for Food, Environmental and Occupational Health & Safety (ANSES), BP 53, 22440 Ploufragan, France; 3 SPF Pig Production and Experimental Unit, French Agency for Food, Environmental and Occupational Health & Safety (ANSES), BP 53, 22440 Ploufragan, France; 4 Université Bretagne Loire, Rennes, France

## Abstract

A transmission experiment involving 5-week-old specific-pathogen-free (SPF) piglets, with (MDA^+^) or without maternally-derived antibodies (MDA^−^), was carried out to evaluate the impact of passive immunity on the transmission of a swine influenza A virus (swIAV). In each group (MDA^+^/MDA^−^), 2 seeders were placed with 4 piglets in direct contact and 5 in indirect contact (3 replicates per group). Serological kinetics (ELISA) and individual viral shedding (RT-PCR) were monitored for 28 days after infection. MDA waning was estimated using a nonlinear mixed-effects model and survival analysis. Differential transmission rates were estimated depending on the piglets’ initial serological status and contact structure (direct contact with pen-mates or indirect airborne contact). The time to MDA waning was 71.3 [52.8–92.1] days on average. The airborne transmission rate was 1.41 [0.64–2.63] per day. The compared shedding pattern between groups showed that MDA^+^ piglets had mainly a reduced susceptibility to infection compared to MDA^−^ piglets. The resulting reproduction number estimated in MDA^+^ piglets (5.8 [1.4–18.9]), although 3 times lower than in MDA^−^ piglets (14.8 [6.4–27.1]), was significantly higher than 1. Such an efficient and extended spread of swIAV at the population scale in the presence of MDAs could contribute to swIAV persistence on farms, given the fact that the period when transmission is expected to be impacted by the presence of MDAs can last up to 10 weeks.

**Electronic supplementary material:**

The online version of this article (doi:10.1186/s13567-016-0365-6) contains supplementary material, which is available to authorized users.

## Introduction

Swine influenza A viruses (swIAVs) cause infections responsible for outbreaks of acute respiratory disease in pigs worldwide [1–3] with a huge morbidity in swine operations [4] and major economic consequences [5, 6] due to growth retardation and complicated bacterial or viral pulmonary troubles [4, 7, 8].

Swine influenza A viruses are polymorphic enveloped single-stranded RNA viruses from the *Orthomyxoviridae* family [4]. The main causative viruses affecting swine production worldwide are H1N1, H1N2 and H3N2 subtypes, which contain genetic components derived from both avian and human influenza viruses leading to different lineages according to their geographical location [9–11]. SwIAVs are shed mainly in respiratory secretions. The general routes of transmission include pig-to-pig contact with infectious pigs, exposure to aerosols or contaminated fomites [12]. However, the contribution of airborne spread on short distance on virus spread within a population has not been quantified to date.

In its classical form, swine flu is known to be responsible for sporadic infections in swine herds, temporarily affecting a huge proportion of the pig population within an infected herd. However, potential persistence between epidemic phases has been increasingly documented in European farrow-to-finish herds [13–16]. This endemic form of swine flu, currently representing up to 40% of the reported cases in France [17], occurs mainly after weaning (21 or 28 days of age), affecting piglets of similar ages in successive batches and favouring constant health disorders on farms. Longitudinal studies have also highlighted the possibility for a piglet to experience simultaneous or consecutive infections by multiple subtypes [15, 16]. This concomitant exposure to different swIAVs can favour genome reassortment, leading to the emergence of novel reassortant viruses potentially more pathogenic for pigs. Public health concerns should also be carefully considered as illustrated by previous pandemic infections, as swIAVs have a zoonotic potential [18].

Several experimental studies were developed to assess the transmission of swIAVs in vaccinated piglets or piglets with passively acquired antibodies resulting from sow vaccination. In 2011, Romagosa et al. [19] showed that vaccination of animals challenged with a homologous influenza virus strain of the American triple reassortant H1N1 lineage completely prevents transmission, whereas only partial protection was observed with heterologous challenge strains. Vaccination was also less effective in pigs challenged with a heterologous H3N2 virus [20] compared to homologous challenged piglets [21]. Besides the impact of their own vaccination, the serological status of piglets at birth is presumed to be of primary importance regarding the spread of influenza among growing pigs. Generally, breeding sows are vaccinated on swine farms at each reproduction cycle [17, 22] to prevent adverse consequences of swIAV infections on reproductive performance. As a result of this regular vaccination of breeding sows, maternally-derived antibodies (MDAs) are transferred to a vast majority of piglets. However, the role of passive immunity in piglets’ early life is controversial [23]. MDAs provide newborn animals with temporary partial protection from infection, but interference between MDAs and post-infectious humoral response was documented as early as 1975 [24]. In addition, a follow-up study carried out in permanently-infected herds revealed that MDA-positive piglets born to routinely-vaccinated dams and affected by an early infection had an impaired post-infectious humoral response [15]. This phenomenon could increase the likelihood of a second swIAV infection [25]. In experimental conditions, previous studies involving strains from American lineages suggested that homologous MDAs derived from vaccination with the same strain as the one used for challenge restrained swIAV transmission [26]. Conversely, piglets with heterologous MDAs (vaccine and challenge strain from different lineages) did not show any significant protection against infection [26, 27]. Other experimental studies have confirmed that MDA-positive piglets are not fully protected, restricting the benefit of MDAs to the reduction of clinical signs only [21, 25, 28].

A unique trivalent inactivated virus vaccine is licensed on European market. It includes three antigens with HA genes belonging to three different viral lineages commonly identified among the swIAVs circulating in European pigs [10, 11, 29]. Quantitative data on epidemiological parameters associated with swIAV infections in the presence of passive immunity in the EU context (different vaccine and challenge strains but from the same lineage) are pivotal to understand the within-herd swIAV endemic feature with systematic infections at similar age in successive batches. Such data could further be used to identify the determinants of swine flu persistence within farrow-to-finish pig herds.

The so-called “protective” effect of MDAs can gather different characteristics including reduced susceptibility to infection and/or reduced shedding in case of infection. The aim of this study was therefore to clarify the impact of MDAs on the dynamics of infection in young piglets both in terms of transmission (direct and indirect) and duration, by quantifying and comparing swIAV spread in piglets in the presence and absence of MDAs under experimental conditions. For that purpose, individual susceptibility to infection and shedding pattern were also investigated to identify the mechanisms explaining the transmission dynamics observed in a population composed of piglets with and without MDAs. Transmission parameters were then estimated according to the contact structure between piglets, including the airborne transmission route, and the initial statuses of piglets regarding the levels of MDAs.

## Materials and methods

### Animals

A transmission experiment was conducted in the ANSES level 3 biosecurity facilities using specific-pathogen-free (SPF) piglets produced in these same facilities. Those SPF pigs have a high health status and beside of being free from major diseases (Classical swine fever, African swine fever, Aujeszky disease, porcine epidemic diarrhea virus, transmissible gastro enteritis virus), they are also free from numerous endemic viruses such as porcine reproductive and respiratory syndrome virus, swIAVs, porcine circovirus type 2, porcine parvovirus and bacteria: *Mycoplasma hyopneumoniae*, *Mycoplasma hyorhinis*, *Actinobacillus pleuropneumoniae*, *Pasteurella multocida*, *Bordetella bronchiseptica*, *Streptococcus suis 2*, and *Haemophilus parasuis*.

SPF dams (gilts) were primo-vaccinated 6 and 3 weeks before insemination with a 2 mL intramuscular injection of an inactivated trivalent H1_av_N1, H1_hu_N2, H3N2 vaccine (Respiporc Flu^®^3, formerly GRIPOVAC^®^3, IDT Biologika GmbH, Dessau-Rosslau, Germany) containing antigens representative of three enzootic lineages circulating in Europe [10, 11], followed by three boosters 6, 3 and 2 weeks before farrowing in order to induce high antibody levels in the colostrum.

Thirty-six SPF piglets born to vaccinated SPF gilts and fed as far as possible by their own dam, constituted a group of piglets with swIAV-specific MDAs (MDA^+^ group), whereas 36 SPF piglets without MDAs (MDA^−^ group), born to unvaccinated SPF gilts, were also used.

### Experimental design

**Figure 1:**
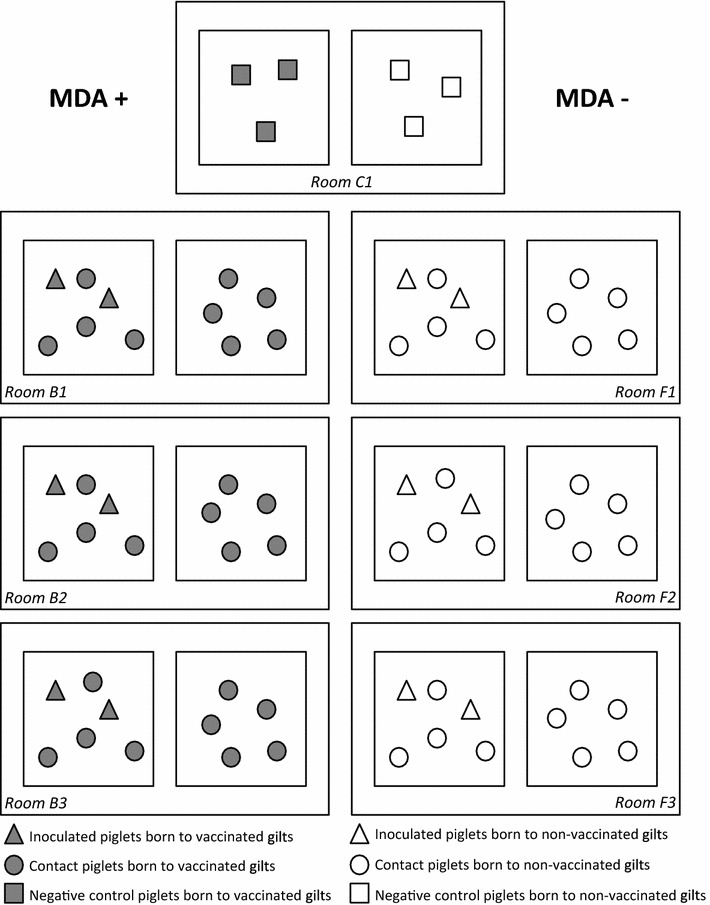
**Description of the experimental design.** Representation of the contact structure of the experiment (inoculated and direct-contact piglets in the pen on the left side of each room; indirect-contact piglets on the right side) for both MDA^+^ group (piglets having maternally-derived antibodies (MDAs); first column, rooms B1, B2 and B3) and MDA^−^ group (piglets without MDAs; second column, rooms F1, F2 and F3). Control piglets (MDA^+^ and MDA^−^) are in room C1.

Thirty-three piglets from each group (MDA^+^ and MDA^−^) were assigned to 6 independent rooms (2 pens per room) according to their serological status (Figure 1). Pen composition was evenly balanced according to the weight, sex, dam’s origin and the 5-week-old MDA level. In each room, 2 seeder piglets were inoculated intratracheally with 10^6^ EID_50_ (Embryonic 50% Infectious Dose) of a European avian-like swine H1N1 (H1_av_N1) virus (A/Sw/Côtes d’Armor/0388/09 strain) from the same genetic lineage than the H1_av_N1 vaccine antigen [10, 11, 29], in a total volume of 5 mL, at 35 days of age (D0). Piglets to be inoculated were gathered in a different room for the inoculation and placed in contact with the other animals 1 day post-inoculation (dpi 1). In each room (3 replicates per group), the 2 seeder piglets were placed in contact with 4 piglets in the same pen to assess transmission through direct contact and 5 piglets were placed in the second pen, 30 cm apart, to assess transmission through indirect contact (no pig-to-pig contact allowed).

The remaining piglets from each group (3 MDA^+^ and 3 MDA^−^ piglets) were placed in 2 different pens in a seventh independent room (Figure 1) and were mock-inoculated [5 mL of minimum essential medium (Eagle, Lonza, Belgium)], constituting the negative control groups. The experiment ended on dpi 28, corresponding to 63 days of age.

The experiment protocol was approved by the Anses/ENVA/UPEC Ethical Committee on animal experiments (agreement #16 with the National committee for Ethics in animal experimentation) and authorized by the French Ministry for Research under the legal notice 11/03/14-17.

### Sampling and laboratory analyses

#### Biosecurity measures

Strict measures were taken to prevent viral transmission through human or fomite contacts. Samples were taken firstly in the indirect-contact pen and secondly in the pen that housed inoculated and direct-contact piglets. Inside the latter, direct-contact piglets were sampled before inoculated piglets. In each room, clothing and footwear were cleaned and gloves changed after each pen visit. Clothing and footwear were changed between each room and a shower was taken when leaving and entering another room. Individual sampling materials were used for each piglet.

#### swIAV serological analyses

Blood samples were collected by jugular vein puncture, using evacuated tubes without an additive (Vacuette, Dutscher SAS, Brumath, France). Vaccinated gilts were sampled 4 and 1 week before, and 3 weeks after, insemination. They were also bled at 7, 5, 3 and 2 weeks before farrowing and 1, 2, 6, 15 and 20 weeks post-farrowing to verify that high antibody levels would be obtained at farrowing and to further monitor serological titres in sera on a long-term basis. In piglets, blood samples were taken at farrowing (34 days before inoculation), 3 days before the trial began and twice a week after infection (dpi 4, 7, 11, 14, 18, 21 and 25). Sera (576 samples) were obtained by blood centrifugation for 5 min at 3500 × *g* and stored at −20 °C. Post-vaccination antibodies, MDAs and post-infection antibodies directed against swIAVs (NP and M antigens) were quantified in sera using LSIVet™ Porcine influenza—Serum ELISA Kit (VETSIV/I, Life Technologies, Courtabœuf, France). Antibody levels are expressed in % IRPC (Relative Index Percent). Although the positive threshold is defined by the manufacturer as 20% IRPC in field conditions, we determined the positive threshold corresponding to our experimental conditions in SPF pigs considering the results from SPF MDA^−^ piglets. Moreover, cross-classified results with HI tests on negative and a range of positive serums were used to define categories of piglets being negative, or with low and high antibody titres. Post-vaccination antibodies in gilts were also titrated in hemagglutination inhibition (HI) tests using virus strains representative for H1_av_N1, H1_hu_N2 and H3N2 lineages as antigens, following the procedure described in [15].

#### swIAV detection and quantification in nasal secretions

To assess viral shedding, nasal swabs (MW951 sent, Virocult®, Corsham, UK) were taken from seeders and direct and indirect contacts from both MDA^+^ and MDA^−^ groups on a daily basis from D0 to dpi 14, and every 2 days thereafter until dpi 28. In control groups, nasal swabs were taken once a week throughout the duration of the experiment. All nasal swab supernatants (1317 samples) were stored at −70 °C until virological analysis.

For swIAV detection, RNA were isolated using NucleoSpin^®^ 8 Virus (Macherey Nagel, Hoerdt, France) and submitted in quadruplicates to high-throughput analyses on LightCycler^®^ 1536 Real-Time PCR Instrument (Roche). Briefly, real-time M gene RT-PCR [30] was conducted in duplex with the amplification of porcine β-actin gene [31] as an internal control. Each replicate comprised 1 µL RNA extract, 0.4 µM and 0.8 µM of M gene forward and reverse primers, respectively, 0.6 µM of β-actin gene primers and 0.25 µM of probes in a final volume of 2 µL. Reverse transcription (RT) was performed for 30 min at 45 °C using GoScript™ RT Mix M700A (Promega) at a 2× final concentration. After an initial activating step of 2 min at 95 °C, PCRs were run using Real Time ready DNA Probes Master Mix (Roche) at a 1× final concentration. Fluorescence data were collected at the end of each of the 40 cycles of 1 s at 95 °C and 30 s at 60 °C. One sample was interpreted positive as soon as M gene was amplified in one replicate out of four.

The swIAV genome was then quantified in selected samples from contact piglets. Five µL of RNA extracts were tested using same primers and probes as above and the GoTaq^®^ Probe 1-Step RT-qPCR System (Promega, Madison, USA) in a total volume of 25 µL (M and β-actin probes at 0.5 µM and 0.3 µM, respectively). RT-PCR (45 °C for 30 s, 95 °C for 2 min, followed by 40 cycles of 95 °C for 15 s and 60 °C for 1 min) was performed in a MX3005P qPCR System (Agilent Technologies, Santa Clara, USA). Serial dilutions of standardized M and β-actin mRNAs were tested similarly to generate standard curves. The swIAV genome amount was expressed as the M gene copy number per 10^4^ copies of β-actin gene.

#### Virus detection in aerosols

To detect swIAV genome in aerosols, air samples were taken in each experimental room housing infected piglets 3 times a week between D-3 and dpi 25 using Coriolis^®^μ microbioal air sampler (Bertin Technologies, St-Quentin en Yvelines, France) (300 L/min, 10 min/room, in 15 mL of 0.005% Triton solution). Collected air samples were concentrated using Amicon^®^ Ultra-15 30 K centrifugal filter devices (Merck Millipore Ltd, Ireland). After centrifugation for 30 min at 3900 × *g*, RNA were purified from 150 µL eluate using RNeasy Mini Kit© (Qiagen GmbH, Hilden, Germany) and 5 µL RNA extract were tested by real-time M gene RT-PCR (LSI VetMAX™ Swine Influenza A-A/H1N1/2009 included, Life Technologies, Lissieu, France) [32]. The conversely cycle threshold 45–Cq values were used to represent a semi-quantitative tendency of the evolution of swIAV genomic load in aerosols.

### Statistical analysis and models

#### Duration of the persistence of maternally-derived antibodies

A nonlinear mixed-effects model (NLME) based on serological data was used to estimate individual parameters governing antibody kinetics in MDA^+^ piglets. The decay of antibody titres was assumed to follow an exponential decrease governed by equation *dA*/*dt* = −*rA*. Thus, the MDA levels depend on the initial level of MDAs *A*_0_ and MDA decay rate *r.* The model describing the serological titre of the individual *i* at observation time *t*_*j*_ (with a constant residual error model) is given by:

$$A_{ij} = A_{0}^{(i)} e^{{ - r_{i} t_{j} }} + a\varepsilon_{ij} ,$$

where *A*_0_^(*i*)^ and *r*_*i*_ are the individual parameters and *ɛ*_*ij*_ a vector of standardised random variables. Individual parameters were assumed to be log-normally distributed, as described in Snoeck et al. [33]. The model for individual parameters is given by:

$$A_{0}^{(i)} = A_{0}^{pop} e^{{\eta_{A}^{(i)} }} \;{\text{and}}\;r_{i} = r_{pop} e^{{\eta_{r}^{(i)} }} ,$$

where *i* denotes the individual, *r*_*pop*_ and *A*_0_^*pop*^   the median decay rate and initial serological level of the global population. Vectors *η*_*A*_^(*i*)^ and *η*_*r*_^(*i*)^ are vectors of random effects assumed to be independent centred Gaussian vectors with variance *Ω*_*A*_ and *Ω*_*r*_ representing inter-individual variability [34]. Population parameters were estimated using MLE by the SAEM algorithm for the hierarchical nonlinear mixed-effects model analysis with Monolix software [35, 36].

Individual parameters were used for long–term projections of individual profiles. A parametric survival analysis with a gamma distribution of survival times was finally carried out to estimate the duration of MDA persistence.

#### Modelling within-host infectious process using viral genome loads quantified in nasal swabs from contact piglets

A model of within-host influenza dynamics [37] was used to compare shedding dynamics in MDA^+^ and MDA^−^ contact piglets based on the target-cell-limited TIV model (susceptible Target cells, productively Infected cells, free Viral particles assessed by viral genome load; [38–40]):

$$\frac{dT}{dt} = - \alpha TV$$

$$\frac{dI}{dt} = \alpha TV - \delta I$$

$$\frac{dV}{dt} = pI - cV.$$

This set of differential equations describes the dynamics of susceptible target cells *T* becoming infected *(I)* at a constant rate *α* by contact with free infectious viral particles *V*. Infected cells *I* produce virus at an average rate *p* per cell until their death at a rate *δ* per day, giving an average lifespan for *I* cells of 1/*δ*. Free viral particles disappear at a clearance rate *c* per day. The effects of the immune response are implicitly included in the *δ* and *c* rates. Parameters governing the differential equations of the model were individually estimated for each group (MDA^+^/MDA^−^) by least squares minimisation using a quasi-Newton algorithm. Based on individual parameters, differences in shedding pattern according to the initial serological status of the animals were assessed by comparing the peak of viral shedding and the global amount of virus shed throughout the shedding period [areas under the fitted curves (AUC)] using ANOVA.

#### Estimation of between-host transmission parameters

The virus transmission process was described by an MSEIR model [41] (Piglets having MDAs—Susceptible—Exposed (i.e. latently infected)—Infectious—Removed), incorporating (i) two different transmission routes according to the contact structure between individuals (direct contact in the same pen or indirect airborne transmission) and (ii) different levels of susceptibility to infection to account for the initial serological status of the piglets (MDA^+^ or MDA^−^). A piglet was considered infectious from the first to the last positive RT-PCR nasal swab and the duration of the shedding period was estimated by parametric survival analysis. In the event of a negative RT-PCR result between two positive results, the piglet was assumed to be infectious at that time. Combining the results of previous experiments [7] and field observations [15], a latency duration of between 0.5 and 1.5 days was selected for contact piglets. Thus, as the exact time contact piglets became infected was not observed due to the latency stage, the interval in which piglets became infected was determined by subtracting 1.5 and 0.5 days (the latency period) from the first positive RT-PCR (*i*_1_) giving an infection interval [*e*_1_, *e*_2_] for each contact-infected piglet calculated as [*i*_1_—1.5, *i*_1_—0.5] [42].

Two transmission routes were considered in this experiment, parameterised by the corresponding transmission rate parameter: (i) direct transmission *β* modelling pig-to-pig contacts between pen-mates and (ii) airborne transmission *β*_*air*_ affecting all animals within a room. Those transmission rates were weighted by a susceptibility-to-infection factor *σ* which accounts for a different susceptibility to infection for piglets in presence of MDAs compared to MDA^−^ piglets (σ = 1 in this latter case). The prevalence of shedding piglets per pen and per room was calculated for each time interval *T* (duration Δ*t*). Assuming that all the shedding animals contribute to infection pressure by the airborne route, the within-room prevalence was considered as an approximation of the virus quantity in the surrounding air. The transmission rates (*β* and *β*_*air*_) represent the number of newly-infected piglets due to one infectious piglet per day. The global force of infection *λ*_*k*_ combines the two viral transmission routes. Thus, a piglet *k* can become infected by direct contact with an infectious pen-mate (transmission rate parameter *β*) or by contact with a contaminated aerosol (parameter *β*_*air*_), weighted by the susceptibility factor *σ* according to its serological status. The global force of infection *λ*_*k*_ for a piglet *k* located in pen *p* and room *r* is thus calculated at time *t* as follows:

$$\lambda_{k} (t) = \beta \frac{{I_{p} (t)}}{{N_{p} - 1}} + \beta_{air} \frac{{I_{r} (t)}}{{N_{r} }}$$

with *I*_*p*_(*t*) and *I*_*r*_(*t*) the (time-dependent) number of infectious piglets in pen *p* or room *r* at time *t*; *N*_*p*_ and *N*_*r*_ the total number of piglets in pen *p* or room *r.*

The probability for each piglet *k* to avoid infection when submitted to the global force of infection *λ*_*k*_ up to time *e*_1_ is $e^{{ - \int_{0}^{{e_{1} ,k}} {\sigma_{k} \lambda_{k} (t)dt} }}$, with *σ*_*k*_ being the susceptibility factor of individual *k*: $$\sigma_{k} = \left\{ {\begin{array}{*{20}c} 1 & {{\text{if}}\quad{\text{MDA}}^{ - } \,{\text{piglet}}} \\ \sigma & {{\text{if}}\quad{\text{MDA}}^{ + } \,{\text{piglet}}} \\ \end{array} } \right.$$. The probability of becoming infected during time interval [*e*_1_, *e*_2_] is therefore $1 - e^{{ - \int_{{e_{1} ,k}}^{{e_{2} ,k}} {\sigma_{k} \lambda_{k} (t)dt} }}$.

The product of all contact-infected piglets gives the full likelihood function *L*:

$$L = \prod\nolimits_{{k \in contact{ - }infected}} {e^{{ - \int_{0}^{{e_{1} ,k}} {\sigma_{k} \lambda_{k} (t)dt} }} *\left( {1 - e^{{ - \int_{{e_{1} ,k}}^{{e_{2} ,k}} {\sigma_{k} \lambda_{k} (t)dt} }} } \right)}$$

The transmission rates (*β* and *β*_*air*_) and the susceptibility factor (*σ*) were then estimated by likelihood maximisation [43]; confidence intervals were derived from the likelihood profile.

The impact of the contact structure and the initial serological status on (i) the shedding start time and (ii) shedding duration was assessed using a semi-parametric Cox model.

## Results

### Serological profiles of gilts and piglets and duration of persistence of maternally-derived antibodies

In gilts, HI tests showed that the primo-vaccination (two injections at week 2 and week 5) led to an increase in titres of HA antibodies directed against the three vaccine antigens, i.e. H1_av_N1, H1_hu_N2 and H3N2 viruses (see Additional file 1). Then, HI titres decreased until the first booster (week 16). The second and the third boosters did not induce any significant new increase in antibody titres but would help maintaining serological titres until farrowing at week 23 (21 weeks after primo-vaccination). Serological analyses using an ELISA targeting all M and NP antibodies displayed the same kinetic pattern for post-vaccination antibodies. Antibodies levels varied from 18 to 28% IRPC at insemination time, 3 weeks after primo-vaccination. Two weeks after the first booster, the levels increased up to 80–90% IRPC, and remained around 70% IRPC after the last boosters until farrowing.

**Figure 2:**
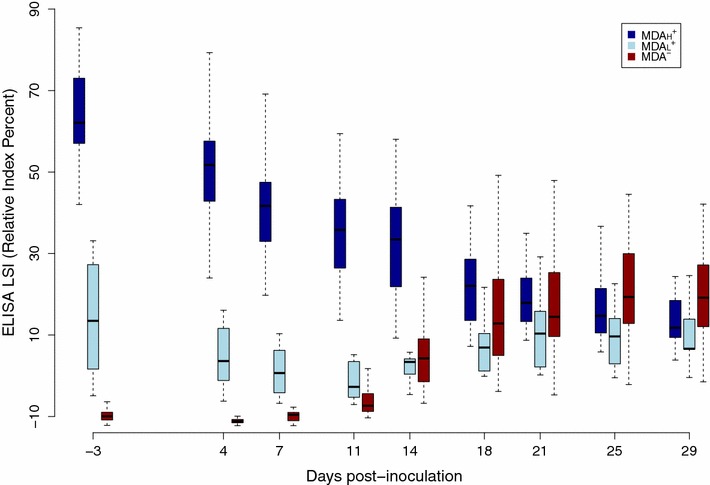
**Swine influenza A serological profiles observed in the different piglet groups.** Serological profiles (ELISA LSI, boxplot) of piglets having high initial antibody levels and showing continuous antibody decay without serological response after infection (MDA_H_^+^, dark blue), piglets having low initial antibody levels and showing moderate serological response after infection (MDA_L_^+^, light blue) and piglets without maternally-derived antibodies and showing a marked serological response after infection (MDA^−^, red).

Considering the similar results obtained in gilts with both methods and the continuous data provided by the ELISA, this assay was used to analyse the serological profiles of the piglets and estimate the duration of MDA persistence. It appeared that the initial antibody levels in MDA^+^ piglets ranged from −6 to 79% IRPC and a limited but non-negligible proportion of piglets were characterized by serological profiles resembling those of MDA^−^ piglets, with a slight increase in antibody levels after infection (Figure 2). In addition, all those piglets with low initial ELISA titres had HI titres below 1:10 (data not shown). Two subgroups were therefore distinguished within the MDA^+^ group, one for piglets with high initial antibody levels (*n* = 26) and the other for low levels (*n* = 7). These two groups are further denoted as MDA_H_^+^ and MDA_L_^+^ respectively. MDA_L_^+^ piglets were characterised by a slight serological response after infection very close to MDA^−^ piglet profiles (*n* = 33) (Figure 2). In the light of the serological response observed in MDA_L_^+^ piglets, the transition from MDA_H_^+^ to MDA_L_^+^ status was selected to be at 10% IRPC. According to this threshold, the mean age of transition (i.e. duration of MDA persistence) estimated by the parametric survival model was 71.3 days [95% confidence interval 52.8–92.1].

### Viral shedding and estimation of transmission parameters

#### Viral shedding

**Figure 3:**
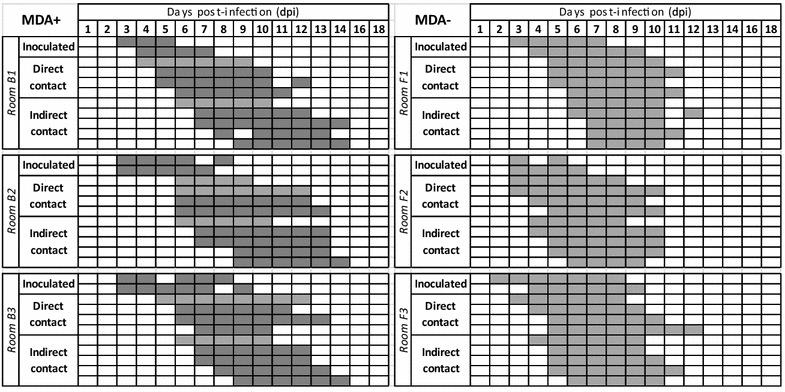
**Individual virological results.** Detection of the influenza A virus genome in nasal secretions of piglets with (first column, rooms B1, B2 and B3) or without passive immunity (second column, rooms F1, F2 and F3), inoculated or in contact (direct or indirect). Grey-tinted squares match positive M gene RT-PCR results (MDA_H_^+^ in dark grey, MDA_L_^+^ and MDA^−^ in light grey).

No viral shedding was detected in the control piglets at any time during the experiment (data not shown). All inoculated piglets began to shed the H1N1 virus between dpi 2 and dpi 4. A similar behaviour was observed in each room: the inoculated piglets began to shed virus first, followed by the direct and indirect contacts (Figure 3). In each room, one or two indirect-contact piglets became infected simultaneously in the second pen through airborne transmission. In the rooms housing MDA^+^ piglets, the first direct- and indirect-contact piglets to become infected were MDA_L_^+^ piglets.

All contact piglets were infected during the experiment with a variable delay according to the contact structure and immune status (MDA^+^ or MDA^−^). The complete infection of all the piglets within the room was longer in MDA^+^ than in MDA^−^ group. MDA^−^ and MDA_L_^+^ contact piglets began to shed the virus more rapidly than MDA_H_^+^ piglets (Hazard Ratio = 3.36 [1.85–6.09]; *p* < 0.05). However, as the individual average duration of shedding was not influenced by the presence of MDAs (HR = 1.39 [0.84–2.30]; *p* = 0.2), the overall average duration of shedding was 6.1 days [5.9–6.4].

#### Within-host infectious process parameters

**Figure 4:**
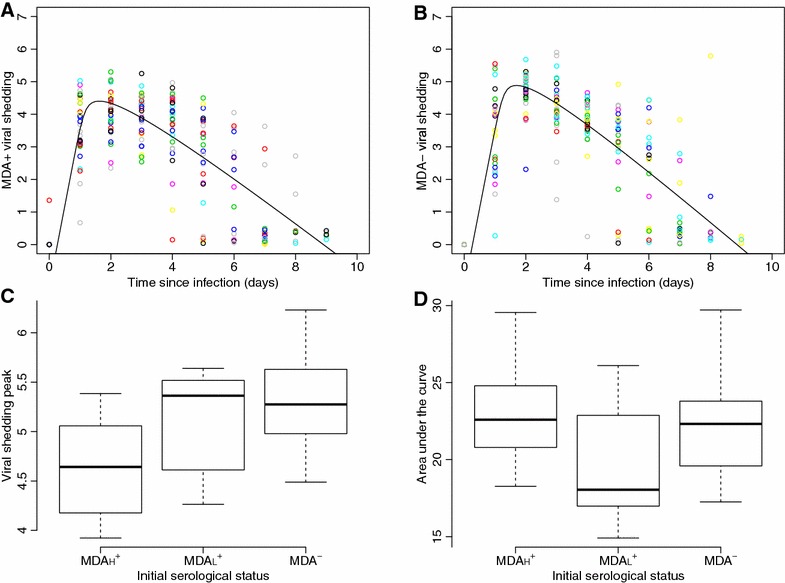
**Shedding profiles of contact piglets and comparison between groups.** Average population profiles of viral shedding in MDA^+^ **(A)** and MDA^−^ **(B)** direct- and indirect-contact piglets; distributions of the viral shedding peak **(C)** and the global amount of viral shedding (AUC, **D**) in MDA_H_^+^, MDA_L_^+^ and MDA^−^ direct- and indirect-contact piglets.

Based on virus detection in nasal secretions, the swIAV genome was quantified in samples from all (MDA^+^ and MDA^−^) direct-contact piglets obtained from dpi 5 to dpi 9, as well as in samples from MDA^+^ and MDA^−^ indirect-contact piglets taken from dpi 6 to dpi 14 and from dpi 4 to dpi 11 respectively. MDA^+^ and MDA^−^ contact piglets exhibited similar individual shedding profiles with a peak on day 2 since infection followed by a rapid decay up to 9 days. The average population profiles are shown in Figures 4A, B (black lines), along with individual data for MDA^+^ and MDA^−^ piglets (dots in Figures 4A, B respectively). The mean population parameters and variation ranges from the ODE model are described in Additional file 2. The characteristics of the virological profiles (peak and global viral shedding) were investigated in the three subgroups previously distinguished using serological data (Figures  4C, D). A slight but significant difference was found between the shedding peaks in MDA_H_^+^ and in MDA^−^ contact piglets, with a difference of 0.7 log_10_ in the average number of viral genome particles between the two groups (4.6 and 5.3 in MDA_H_^+^ and in MDA^−^ piglets respectively; *p* < 0.001). The peak in MDA_L_^+^ piglets was in the same range as the MDA^−^ group (*p* = 0.08). No significant difference was found between the average global amount of virus shed by the three subpopulations (22.5, 19.8 and 22.2 for AUC in MDA_H_^+^, MDA_L_^+^ and MDA^−^ groups respectively; *p* = 0.18).

#### Between-host transmission parameters

Considering the above mentioned diversity in serological profiles, three models weighting the susceptibility to infection according to the MDA levels of the piglets were tested for transmission parameter estimation: (i) three factors of susceptibility were estimated accounting for serological profile diversity (i.e. high level, low level, and absence of MDAs) (*σ*_*H*_ ≠ *σ*_*L*_, model 1); (ii) owing to the limited number of MDA_L_^+^ piglets (*n* = 7) and the similarity with MDA^−^ piglets of their post-infectious serological profile, the same susceptibility to infection were assumed for these both groups (*σ*_*L*_ = 1, model 2); (iii) a unique factor of susceptibility was estimated for all MDA^+^ piglets, the serological profile diversity between MDA_H_^+^ and MDA_L_^+^ piglets was not taking into account (*σ*_*H*_ = *σ*_*L*_, model 3). The parameters estimated for each model as well as the criteria of model quality are summarized in Table 1.

**Table 1:** **Estimation of the transmission parameters [95 % Confidence Interval] for MDA**_**H**_^**+**^ **(** ***n*** **= 26),** **MDA**_**L**_^**+**^ **(** ***n*** **= 7) and MDA** ^**−**^ **(** ***n*** **= 33)** **piglets**

	Transmission rates		Susceptibility factors		BIC
	*β (days* ^−*1*^ *)*	*β*_*air*_ *(days* ^−*1*^ *)*	*σ*_*H*_	*σ*_*L*_	
Model 1	2.48 [1.08–4.56]	1.43 [0.64–2.74]	0.38 [0.20–0.72]	0.92 [0.35–2.18]	127.3
Model 2	2.43 [1.09–4.23]	1.41 [0.64–2.63]	0.39 [0.21–0.70]	–	116.2
Model 3	2.60 [1.20–4.69]	1.35 [0.60–2.62]	0.45^a^ [0.25–0.83]		119.4

Overall population estimate applying to MDA_H_^+^ and MDA_L_^+^ piglets; BIC: Bayesian Information Criteria

With *β* being the direct transmission rate, *β*_*air*_ the airborne transmission rate, *σ*_*H*_ the susceptibility factor for MDA_H_^+^ piglets and *σ*_*L*_ the susceptibility factor for MDA_L_^+^ piglets.

Model 1: different susceptibility factors for MDA_H_^+^, MDA_L_^+^ and MDA^−^ piglets (*σ*_*H*_ ≠ *σ*_*L*_ and *σ*_*H*,*L*_ < 1).

Model 2: same susceptibility factor for MDA_L_^+^ and MDA^−^ piglets (*σ*_*L*_ = 1 and *σ*_*H*_ < 1).

Model 3: same susceptibility factor for MDA_H_^+^ and MDA_L_^+^ piglets (*σ*_*H*_ = *σ*_*L*_ et *σ*_*H*,*L*_ < 1).

The BIC (Bayesian Information Criteria) highlighted the relevance of estimating the same susceptibility to infection for MDA_L_^+^ and MDA^−^ piglets (BIC_model-2_ = 116.2). Under experimental conditions, a fully-susceptible piglet (MDA^−^ or MDA_L_^+^ piglet) was able to infect 2.43 piglets per day by direct contact (*β* = 2.43 [1.09–4.23]), which was almost threefold higher than MDA_H_^+^ piglets (*σ*_*H*_ = 0.39 [0.21–0.70], *σ*_*H*_*β* = 0.95 [0.23–2.96]). Within a room, 1.41 piglets became infected via the air per day (airborne transmission rate *β*_*air*_ = 1.41 [0.64–2.63]).

A combination of the estimated transmission rates and a shedding period of 6.1 days [5.9–6.4] was used to estimate reproduction numbers: MDA_H_^+^ piglets were infected despite the presence of MDAs and could transmit the virus on average to 5.8 piglets [1.4–18.9] during their entire shedding period whereas a fully-susceptible piglet could infect 14.8 [6.4–27.1] piglets during the same time (Table 2).

**Table 2:** **Estimation of the reproduction numbers R**_**0**_ **for MDA** ^**+**^ **and MDA** ^**−**^ **piglets using the best model (model 2)**

	Shedding period *(days)*	Direct transmission rate β *(days* ^−*1*^ *)*	Susceptibility factor σ	R_0_
MDA^+^ piglets	6.1 [5.9–6.4]^a^	2.43 [1.09–4.23]^a^	0.39 [0.21–0.70]	14.8 [6.4–27.1]
MDA^−^ piglets			**–**	5.8 [1.4–18.9]

^a^ Overall population estimate applying to both groups.

**Figure 5:**
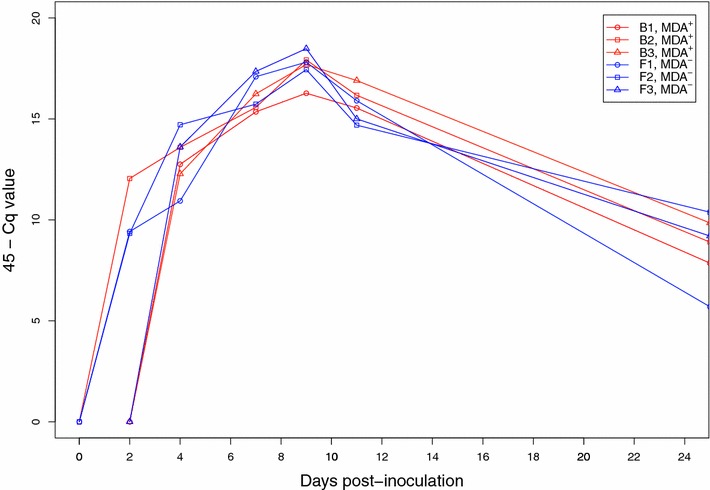
**Viral genome loads in aerosols.** Deduced from M gene RT-PCR analyses (45—Cq value) performed on air samples taken in each room (rooms B1, B2 and B3 correspond to rooms housing piglets with maternally-derived antibodies (MDAs); rooms F1, F2 and F3 to piglets without MDAs) during the animal experiment.

No difference was observed between MDA^+^ and MDA^−^ piglet groups as regards the course of the estimated swIAV genome load in air samples. In each room, the virus genome load peaked at dpi 9, after which a significant amount of virus genome being detected in the air 13 days after the last shedding piglets (Figure 5). Considering a 1-day latency period, the first piglets became infected in the indirect-contact pen on dpi 5 and dpi 3 in the MDA^+^ and the MDA^−^ groups respectively, when there was a substantial amount of virus genome in the air.

## Discussion

Understanding the epidemiological processes responsible for endemic swIAV persistence within pig herds requires a quantitative approach to viral transmission parameters within a pig population. Data from field studies have suggested an interaction between the immune status of animals at the time of infection and viral spread between batches, leading to persistence of infection on farms [15, 16]. The transmission experiment herein described was designed to estimate transmission parameters in controlled conditions and compare these estimates for two populations differing only by their maternally derived immune status. Such references in terms of quantitative estimates of transmission parameters are required to assess the consequences of swIAV infections in young animals still having MDAs, and evaluate assumptions related to factors increasing the likelihood of viral persistence within a population. Two complementary approaches were thus developed, considering on the one hand within-host models to analyse individual serological and virological data, and on the other, between-host models to evaluate the transmission of swIAVs in a structured population.

In this study, two different viral transmission routes were considered within the same room: (i) direct transmission, which can only occur between pen-mates through pig-to-pig contact and (ii) indirect airborne transmission, which can occur between all the pigs in a room, including pen-mates. Although carried out in experimental conditions, the design was developed to represent a contact structure close to field condition, accounting for transmission between pen-mates and neighbouring pens.

The individual profiles of maternal antibody decay in piglets born to vaccinated gilts led to an estimated 71.3 days of MDA persistence [52.8 – 92.1]. A transition to a “more-susceptible-to-infection” status around 10 weeks seems realistic for SPF piglets born to multi-vaccinated gilts. Indeed, in field conditions, MDAs were shown to persist for 11 weeks on average [15]. In experimental conditions, piglets born to conventional vaccinated sows showed MDA persistence ranging from 8 to 13 weeks of age [44, 45]. Like in previous studies, the current experiment revealed heterogeneity between individual profiles. This could be linked to dam antibody levels and/or uneven colostrum intake by piglets. MDA concentration in the colostrum has been shown to decline rapidly in the first 24 h [46]. An appropriate colostrum intake in the first hours of life is known to be crucial for supplying newborn piglets with high levels of MDAs. In our study, 7 piglets born to an aggressive gilt had to be cross-fostered. Born a few hours after the others, these piglets probably had limited access to rich colostrum [46]. Four of the 7 cross-fostered piglets were incidentally in the MDA_L_^+^-piglet group.

The observed heterogeneity in serological status at time of infection was strongly associated with the pattern of post-infectious serological response. As described previously [15, 25], no detectable serological response after infection was observed in MDA_H_^+^ piglets, while piglets with low antibody levels at the time of infection (MDA_L_^+^) showed a moderate serological response. The transmission characteristics of MDA_L_^+^ piglets were also significantly different from those of MDA_H_^+^ piglets, contributing more to the transmission process. Differences in the viral kinetics of these two groups were also highlighted by a within-host modelling approach which revealed a higher shedding peak in MDA_L_^+^ piglets than in MDA_H_^+^ animals. However, the global within-host infectious process and the amount of virus genome shed by infected individuals were not found to differ significantly in these two sub-populations. It suggests that the presence of maternally-derived antibodies did not strongly affect the individual shedding pattern once an animal was infected. Therefore, differences between MDA_H_^+^ and MDA_L_^+^ piglets are more likely related to a higher susceptibility to infection of piglets having low antibody levels after weaning. Consistent with this assumption, MDA_L_^+^ piglets were the first to be infected in the room housing piglets born to vaccinated dams. It therefore reinforces the relevance of accounting for heterogeneity rather than considering all piglets born to vaccinated dams as a whole. In contrast, shedding patterns and serological responses observed in MDA_L_^+^ piglets are similar to those of MDA^−^ piglets, emphasising the advantage in considering the same susceptibility factor for these two subgroups.

The estimates in MDA^+^ (0.95 [0.23–2.96]) and in MDA^-^ piglets (2.43 [1.09–4.23]) agree with the literature [15, 19, 26]. Recently, Allerson et al. [26] estimated the transmission rates of a H1N1 strain of American lineage at 1.74 [1.18–2.46] and 2.18 [1.47–3.10] in conventional piglets born to vaccinated (distinct lineage between the vaccine and the challenge strain) and non-vaccinated sows, respectively. A higher homology between the vaccinal and the inoculated strains (HA genes belonging to the same genetic lineage in our experiment versus different clades in Allerson’s study) could have induced a higher protection in our experimental conditions. Also, we can not exclude differences in terms of transmission related to the status of the animals (SPF piglets in our experiment versus conventional piglets in Allerson’s study) and/or the design of the experiment, which in our case considers both direct and indirect transmission. The European avian-like swine H1N1 (H1_av_N1) virus used in the present experiment is the most frequently detected swIAV in Europe [10] but it would be interesting to reproduce the present experiment with swIAVs from other enzootic lineages included in the vaccine to compare transmission parameters.

Inoculated piglets began viral shedding between dpi 2 and dpi 4. Previous data from published studies corroborate these results [7, 19]. Intratracheal inoculation potentially differs from a natural infection process by aerosols which could be faster. In field conditions, a latency period of 0.5 to 1.5 days has been estimated [15]. The delay between the first viral shedding in inoculated and contact piglets was sometimes within one day in our experiment. A latency period varying from 0.5 to 1.5 days was therefore selected to account for all these components.

To our knowledge, this is the first time that the airborne transmission rate of swIAV has been quantified. Although several studies have investigated this topic, focussing either on the relationship between the number of infected pigs and viral presence in the air [47] or the quantification of the virus in the air [48], no transmission parameters have been estimated to date. The airborne virus transmission rate estimated in our study revealed that 1.41 piglets became infected per day via the air and at a lower rate in MDA^+^ piglets because of reduced susceptibility. Each indirect-contact pen became infected in all the rooms, demonstrating the need to account for the airborne component in the infection process so as to accurately understand the spread and persistence of swine flu. Indeed, compared to field conditions with several 100 piglets per room, a limited number of infectious piglets were required to infect room-mates by the airborne route in this experimental study. The ventilation system in the ANSES experimental facilities is also more efficient than on conventional pig farms, as a smaller volume of air is renewed more frequently. This parameter could have induced an underestimation of airborne transmission compared to field conditions. The swIAV genome was furthermore detected in the air of the room up to 13 days after the last shedding piglet was detected, highlighting the persistence of viral RNA in aerosols and the importance of considering this issue in endemic swIAV within-herd management. Airborne swIAV detection has been also reported previously in the environment of infected MDA^+^ pigs [49]. From our results, the observed increase in the deduced amount of virus genome in the air and individual viral shedding corresponds also to the point where the incidence of infection is the higher within the pen.

The duration of the shedding period estimated in the present study is longer than previously published experimental data [26] but shorter than field-based estimations [15]. The differences could be linked to the status of the piglets (SPF piglets in the present case) as well as the assessment and characteristics of influenza outbreaks described in field conditions. In the latter case, the length of the shedding period was also estimated on the basis of M gene RT-PCR results which do not discriminate between subtypes, but characterization of the isolates showed a frequent observation of the co-circulation of 2 subtypes slightly delayed over time, which artificially increased the estimated shedding period based on M gene RT-PCR results.

Similarly to Lange et al. [50], a concise global shedding period was observed at group scale in MDA^−^ contact piglets. Although displaying maternal immunity against the H1N1 subtype, piglets with high initial antibody levels (MDA_H_^+^) revealed a high potential for viral spread. The reproduction number (i.e. smaller than that for MDA^−^ piglets but significantly higher than 1) led to an extended total duration of the infectious process at the scale of a maternally-immune pig population. Transposed to field conditions, this extended infectious period could foster within-herd viral persistence through the permanent exposure of incoming susceptible piglets to shedding animals from other batches [15, 16]. This assumption would have to be evaluated in a simulation study. Likewise, a longer period with shedding piglets at herd level increases the probability of concomitant multiple-strain circulation and thus generation of reassortant viruses [15].

In this experiment, piglets were inoculated at 35 days of age, corresponding to what has been frequently observed on pig farms affected by endemic influenza persistence when it strikes piglets after weaning [15]. At that age, piglets still have high levels of MDAs, so transmission was evaluated in a maximised situation. According to our estimate of the duration of MDA persistence, the interval when transmission is expected to be impacted by the presence of MDAs is quite large (up to 10 weeks). This would be consistent with current field observations, which suggest a high prevalence of endemic swIAV situations in farrow-to-finish farms. It also raises the question of the effect of regular sow herd vaccination before farrowing in this context, which would boost antibody production in sows and increase the levels of MDAs received by piglets through colostrum. Indeed, based on the serological results of the gilts, we can see that the primo-vaccination induced a moderate serological response, while the first booster carried out 13 weeks later (i.e. a few weeks before farrowing) induced a dramatic increase in antibody levels which were still detectable 4 months after the last booster (around the next expected farrowing time).

The spread of influenza virus is modified by piglets’ maternally-derived immune status at the time of infection and inter-piglet contact structure. Although the presence of MDAs in weaned piglets significantly reduces swIAV transmission, the reproduction number is significantly higher than 1, highlighting the limited protection conferred by MDAs against the spread of influenza virus. Adverse effects are observed: the dissemination process is slower compared to piglets without passive immunity, fostering the presence of shedding animals over a longer period at population scale, which could enhance swine flu within-herd persistence. Thus, it appears crucial to account for the maternally-derived immune status of the animals and the contact structure (direct or by airborne route) between weaned piglets in order to identify control measures designed to prevent endemic persistence of swIAV infections in pig herds. Airborne transmission was also identified as a key point, highlighting the need to consider infectious aerosols as a major issue possibly participating to within-herd swIAV spread and persistence.

## Additional files

10.1186/s13567-016-0365-6 **Swine influenza A serological profiles of vaccinated gilts.** Serological profiles (HI tests and ELISA LSI) of the vaccinated dams starting from first injection (Primo vaccination at weeks 2 and 5, 1st, 2nd and 3rd boosters at weeks 16, 20 and 21 respectively).
10.1186/s13567-016-0365-6 **Within-host model parameters estimated from individual viral shedding kinetics in contact pigs.** The given values correspond to the mean and the 95% confidence interval [95% CI] of estimated individual parameters.

## References

[1] Crisci E, Mussá T, Fraile L, Montoya M (2013). Review: influenza virus in pigs. Mol Immunol.

[2] Loeffen WL, Kamp EM, Stockhofe-Zurwieden N, van Nieuwstadt AP, Bongers JH, Hunneman WA, Elbers AR, Baars J, Nell T, van Zijderveld FG (1999). Survey of infectious agents involved in acute respiratory disease in finishing pigs. Vet Rec.

[3] Terebuh P, Olsen CW, Wright J, Klimov A, Karasin A, Todd K, Zhou H, Hall H, Xu X, Kniffen T, Madsen D, Garten R, Bridges CB (2010). Transmission of influenza A viruses between pigs and people, Iowa, 2002–2004. Influenza Other Respir Viruses.

[4] Van Reeth K, Brown IH, Olsen CW (2012) Influenza virus. In: Zimmerman JJ, Karriker LA, Ramirez A, Schwartz KJ, Stevenson GW (eds) Diseases of Swine 10^th^. Wiley-Blackwell, p 557–571. http://eu.wiley.com/WileyCDA/WileyTitle/productCd-081382267X.html

[5] Bennett R, Ijpelaar J (2005). Updated estimates of the costs associated with thirty four endemic livestock diseases in Great Britain: a note. J Agr Econ.

[6] Brons N, Neto R, Vila T, Pasini M, Joisel F (2011) Outbreak of swine influenza, subtype H1N2: a case report and its financial consequences, In: Proceedings of the 6th international symposium on emerging and re-emerging pig diseases, Barcelona, Spain, p 271

[7] Deblanc C, Gorin S, Quéguiner S, Gautier-Bouchardon AV, Ferré S, Amenna N, Cariolet R, Simon G (2012). Pre-infection of pigs with *Mycoplasma hyopneumoniae* modifies outcomes of infection with European swine influenza virus of H1N1, but not H1N2, subtype. Vet Microbiol.

[8] Fablet C, Marois-Créhan C, Simon G, Grasland B, Jestin A, Kobisch M, Madec F, Rose N (2012). Infectious agents associated with respiratory diseases in 125 farrow-to-finish pig herds: a cross-sectional study. Vet Microbiol.

[9] Brown IH (2013). History and epidemiology of swine influenza in europe. Curr Top Microbiol.

[10] Simon G, Larsen LE, Durrwald R, Foni E, Harder T, Van Reeth K, Markowska-Daniel I, Reid SM, Dan A, Maldonado J, Huovilainen A, Billinis C, Davidson I, Aguero M, Vila T, Herve S, Breum SO, Chiapponi C, Urbaniak K, Kyriakis CS, Brown IH, Loeffen W (2014). European surveillance network for influenza in pigs: surveillance programs, diagnostic tools and Swine influenza virus subtypes identified in 14 European countries from 2010 to 2013. PLoS One.

[11] Watson SJ, Langat P, Reid SM, Lam TT, Cotten M, Kelly M, Van Reeth K, Qiu Y, Simon G, Bonin E, Foni E, Chiapponi C, Larsen L, Hjulsager C, Markowska-Daniel I, Urbaniak K, Durrwald R, Schlegel M, Huovilainen A, Davidson I, Dan A, Loeffen W, Edwards S, Bublot M, Vila T, Maldonado J, Valls L, Brown IH, Pybus OG, Kellam P (2015). Molecular epidemiology and evolution of influenza viruses circulating within European swine between 2009 and 2013. J Virol.

[12] Tellier R (2006). Review of aerosol transmission of influenza A virus. Emerg Infect Dis.

[13] Loeffen WLA, Hunneman WA, Quak J, Verheijden JHM, Stegeman JA (2009). Population dynamics of swine influenza virus in farrow-to-finish and specialised finishing herds in the Netherlands. Vet Microbiol.

[14] Madec F, Gourreau JM, Kaiser C, Le Dantec J, Vannier P, Aymard M (1985). The persistence of activity of H1N1 (swine) influenza virus in pig breeding units during non-epidemic phases. Comp Immunol Microbiol Infect Dis.

[15] Rose N, Hervé S, Eveno E, Barbier N, Eono F, Dorenlor V, Andraud M, Camsusou C, Madec F, Simon G (2013). Dynamics of influenza a virus infections in permanently infected pig farms: Evidence of recurrent infections, circulation of several swine influenza viruses and reassortment events. Vet Res.

[16] Simon-Grife M, Martin-Valls GE, Vilar MJ, Busquets N, Mora-Salvatierra M, Bestebroer TM, Fouchier RA, Martin M, Mateu E, Casal J (2012). Swine influenza virus infection dynamics in two pig farms; results of a longitudinal assessment. Vet Res.

[17] Hervé S, Garin E, Rose N, Marcé C, Simon G (2014). French network for the surveillance of influenza viruses in pigs (Résavip)—results of the first three years of operation. Bull Epidémiol Santé Anim Alim Anses-DGAl.

[18] Vincent A, Awada L, Brown I, Chen H, Claes F, Dauphin G, Donis R, Culhane M, Hamilton K, Lewis N, Mumford E, Nguyen T, Parchariyanon S, Pasick J, Pavade G, Pereda A, Peiris M, Saito T, Swenson S, Van Reeth K, Webby R, Wong F, Ciacci-Zanella J (2013). Review of influenza A virus in swine worldwide: a call for increased surveillance and research. Zoonoses Public Hlth.

[19] Romagosa A, Allerson M, Gramer M, Joo H, Deen J, Detmer S, Torremorell M (2011). Vaccination of influenza a virus decreases transmission rates in pigs. Vet Res.

[20] Loving CL, Lager KM, Vincent AL, Brockmeier SL, Gauger PC, Anderson TK, Kitikoon P, Perez DR, Kehrli ME (2013). Efficacy in pigs of inactivated and live attenuated influenza virus vaccines against infection and transmission of an emerging H3N2 similar to the 2011–2012 H3N2v. J Virol.

[21] Bosworth B, Erdman MM, Stine DL, Harris I, Irwin C, Jens M, Loynachan A, Kamrud K, Harris DL (2010). Replicon particle vaccine protects swine against influenza. Comp Immunol Microbiol Infect Dis.

[22] Vincent AL, Ma W, Lager KM, Janke BH, Richt JA (2008) Chapter 3 Swine influenza viruses. A North American perspective, advances in virus research. Elsevier, p 127–154. https://www.ars.usda.gov/2009H1N1/pdfs/VincentAdVirResv72p127y08.pdf 10.1016/S0065-3527(08)00403-X 19081490

[23] Salmon H, Berri M, Gerdts V, Meurens F (2009). Humoral and cellular factors of maternal immunity in swine. Dev Comp Immunol.

[24] Renshaw HW (1975). Influence of antibody mediated immune suppression on clinical, viral, and immune responses to swine influenza infection. Am J Vet Res.

[25] Loeffen WLA, Heinen PP, Bianchi ATJ, Hunneman WA, Verheijden JHM (2003). Effect of maternally derived antibodies on the clinical signs and immune response in pigs after primary and secondary infection with an influenza H1N1 virus. Vet Immunol Immunop.

[26] Allerson M, Deen J, Detmer SE, Gramer MR, Joo HS, Romagosa A, Torremorell M (2013). The impact of maternally derived immunity on influenza A virus transmission in neonatal pig populations. Vaccine.

[27] Rajao DS, Sandbulte MR, Gauger PC, Kitikoon P, Platt R, Roth JA, Perez DR, Loving CL, Vincent AL (2016). Heterologous challenge in the presence of maternally-derived antibodies results in vaccine-associated enhanced respiratory disease in weaned piglets. Virology.

[28] Kitikoon P, Nilubol D, Erickson BJ, Janke BH, Hoover TC, Sornsen SA, Thacker EL (2006). The immune response and maternal antibody interference to a heterologous H1N1 swine influenza virus infection following vaccination. Vet Immunol Immunop.

[29] Lewis NS, Russell CA, Langat P, Anderson TK, Berger K, Bielejec F, Burke DF, Dudas G, Fonville JM, Fouchier RA, Kellam P, Koel BF, Lemey P, Nguyen T, Nuansrichy B, Peiris JM, Saito T, Simon G, Skepner E, Takemae N, Webby RJ, VanReeth K, Brookes SM, Larsen L, Watson SJ, Brown IH, Vincent AL (2016). The global antigenic diversity of swine influenza A viruses. eLife.

[30] Weingartl HM, Berhane Y, Hisanaga T, Neufeld J, Kehler H, Emburry-Hyatt C, Hooper-McGreevy K, Kasloff S, Dalman B, Bystrom J, Alexandersen S, Li Y, Pasick J (2010). Genetic and pathobiologic characterization of pandemic H1N1 2009 influenza viruses from a naturally infected swine herd. J Virol.

[31] Duvigneau JC, Hartl RT, Groiss S, Gemeiner M (2005). Quantitative simultaneous multiplex real-time PCR for the detection of porcine cytokines. J Immunol Methods.

[32] Pol F, Quéguiner S, Gorin S, Deblanc C, Simon G (2011). Validation of commercial real-time RT-PCR kits for detection of influenza A viruses in porcine samples and differentiation of pandemic (H1N1) 2009 virus in pigs. J Virol.

[33] Snoeck E, Chanu P, Lavielle M, Jacqmin P, Jonsson EN, Jorga K, Goggin T, Grippo J, Jumbe NL, Frey N (2010). A comprehensive hepatitis C viral kinetic model explaining cure. Clin Pharmacol Ther.

[34] Andraud M, Casas M, Pavio N, Rose N (2014). Early-life hepatitis e infection in pigs: the importance of maternally-derived antibodies. PLoS One.

[35] Lavielle M, Mentre F (2007). Estimation of population pharmacokinetic parameters of saquinavir in HIV patients with the MONOLIX software. J Pharmacokinet Phar.

[36] Lixoft, modelling and simulation for drug development. http://monolix.lixoft.com/ Accessed 16 Dec 2015

[37] Beauchemin CA, Handel A (2011). A review of mathematical models of influenza A infections within a host or cell culture: lessons learned and challenges ahead. BMC Public Health.

[38] Baccam P, Beauchemin C, Macken CA, Hayden FG, Perelson AS (2006). Kinetics of influenza A virus infection in humans. J Virol.

[39] Canini L, Carrat F (2011). Population modeling of influenza A/H1N1 virus kinetics and symptom dynamics. J Virol.

[40] Saenz RA, Quinlivan M, Elton D, Macrae S, Blunden AS, Mumford JA, Daly JM, Digard P, Cullinane A, Grenfell BT, McCauley JW, Wood JL, Gog JR (2010). Dynamics of influenza virus infection and pathology. J Virol.

[41] Hens N, Shkedy Z, Aerts M, Faes C, Van Damme P, Beutels P (2012). Modeling Infectious Disease Parameters Based on Serological and Social Contact Data A Modern Statistical Perspective.

[42] Weesendorp E, Backer J, Loeffen W (2014). Quantification of different classical swine fever virus transmission routes within a single compartment. Vet Microbiol.

[43] Andraud M, Grasland B, Durand B, Cariolet R, Jestin A, Madec F, Rose N (2008). Quantification of porcine circovirus type 2 (PCV-2) within- and between-pen transmission in pigs. Vet Res.

[44] Markowska-Daniel I, Pomorska-Mól M, Pejsak Z (2011). The influence of age and maternal antibodies on the postvaccinal response against swine influenza viruses in pigs. Vet Immunol Immunop.

[45] Vincent AL, Ma W, Lager KM, Richt JA, Janke BH, Sandbulte MR, Gauger PC, Loving CL, Webby RJ, García-Sastre A (2012). Live attenuated influenza vaccine provides superior protection from heterologous infection in pigs with maternal antibodies without inducing vaccine-associated enhanced respiratory disease. J Virol.

[46] Rooke JA, Bland IM (2002). The acquisition of passive immunity in the new-born piglet. Livest Prod Sci.

[47] Corzo CA, Romagosa A, Dee SA, Gramer MR, Morrison RB, Torremorell M (2013). Relationship between airborne detection of influenza A virus and the number of infected pigs. Vet J.

[48] Corzo CA, Culhane M, Dee S, Morrison RB, Torremorell M (2013). Airborne detection and quantification of swine influenza a virus in air samples collected inside, outside and downwind from swine barns. PLoS One.

[49] Corzo CA, Allerson M, Gramer M, Morrison RB, Torremorell M (2014). Detection of airborne influenza a virus in experimentally infected pigs with maternally derived antibodies. Transbound Emerg Dis.

[50] Lange E, Kalthoff D, Blohm U, Teifke JP, Breithaupt A, Maresch C, Starick E, Fereidouni S, Hoffmann B, Mettenleiter TC, Beer M, Vahlenkamp TW (2009). Pathogenesis and transmission of the novel swine-origin influenza virus A/H1N1 after experimental infection of pigs. J Gen Virol.

