# Erratum to: Maternally-derived antibodies do not prevent transmission of swine influenza A virus between pigs

**DOI:** 10.1186/s13567-016-0382-5

**Published:** 2016-09-21

**Authors:** Charlie Cador, Séverine Hervé, Mathieu Andraud, Stéphane Gorin, Frédéric Paboeuf, Nicolas Barbier, Stéphane Quéguiner, Céline Deblanc, Gaëlle Simon, Nicolas Rose

**Affiliations:** 1 Swine Epidemiology and Welfare Research Unit, French Agency for Food, Environmental and Occupational Health & Safety (ANSES), BP 53, 22440 Ploufragan, France; 2 Swine Virology Immunology Research Unit, French Agency for Food, Environmental and Occupational Health & Safety (ANSES), BP 53, 22440 Ploufragan, France; 3 SPF Pig Production and Experimental Unit, French Agency for Food, Environmental and Occupational Health & Safety (ANSES), BP 53, 22440 Ploufragan, France; 4 Université Bretagne Loire, Rennes, France

## Erratum to: Vet Res (2016) 47:86 DOI 10.1186/s13567-016-0365-6

After publication of the original article [1], the authors noticed an error in Table 2 in the “Results” section. In the last column the R_0_ estimated values have been reversed between groups. Consistently with the text, R_0_ is 5.8 [1.4–18.9] for MDA^+^ piglets (first row) and 14.8 [6.4–27.1] for MDA^−^ piglets (second row). The corrected version of Table 2 is included in this erratum.

**Table 2:** **Estimation of the reproduction numbers R**_**0**_ **for MDA** ^**+**^ **and MDA** ^**−**^ **piglets using the best model (model 2)**

	Shedding period (days)	Direct transmission rate β (days^−1^)	Susceptibility factor σ	R_0_
MDA^+^ piglets			0.39 [0.21–0.70]	5.8 [1.4–18.9]
	6.1 [5.9 – 6.4]^a^	2.43 [1.09 – 4.23]^a^		
MDA^−^ piglets			–	14.8 [6.4–27.1]

^a^Overall population estimate applying to both groups.
