# Supplementary figures and images for: Maternally-derived antibodies do not prevent transmission of swine influenza A virus between pigs
Source: Vet Res. 2016 Aug 17;47:86. doi: 10.1186/s13567-016-0365-6 (PMC4988049; doi:10.1186/s13567-016-0365-6)

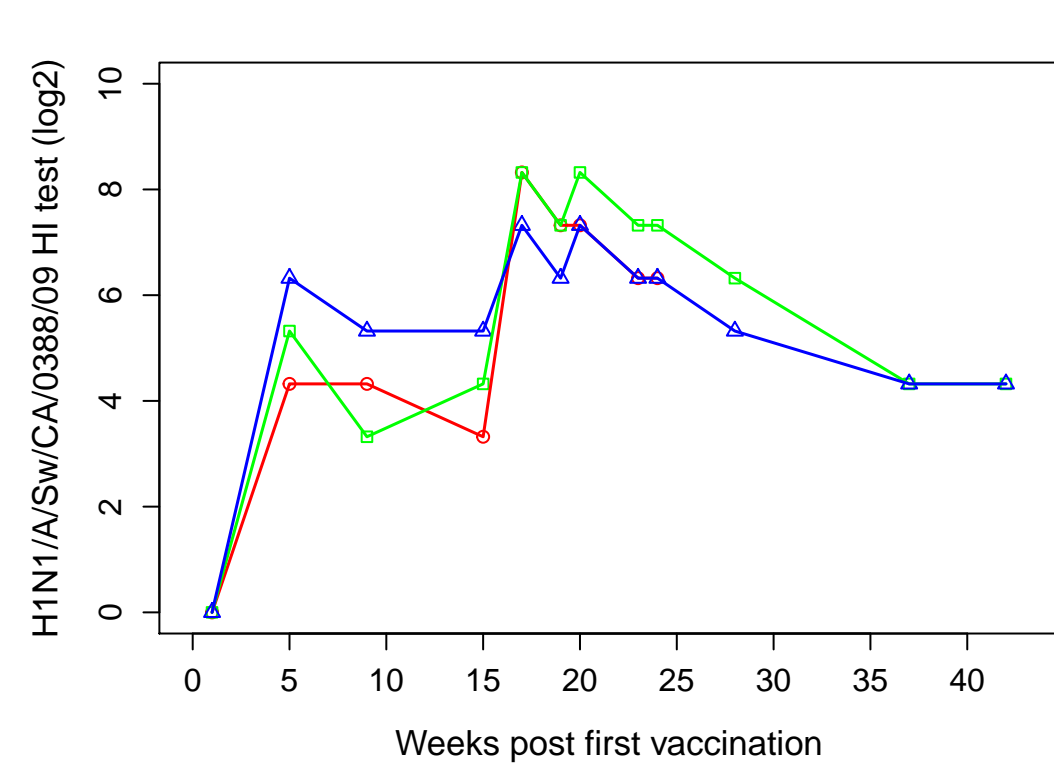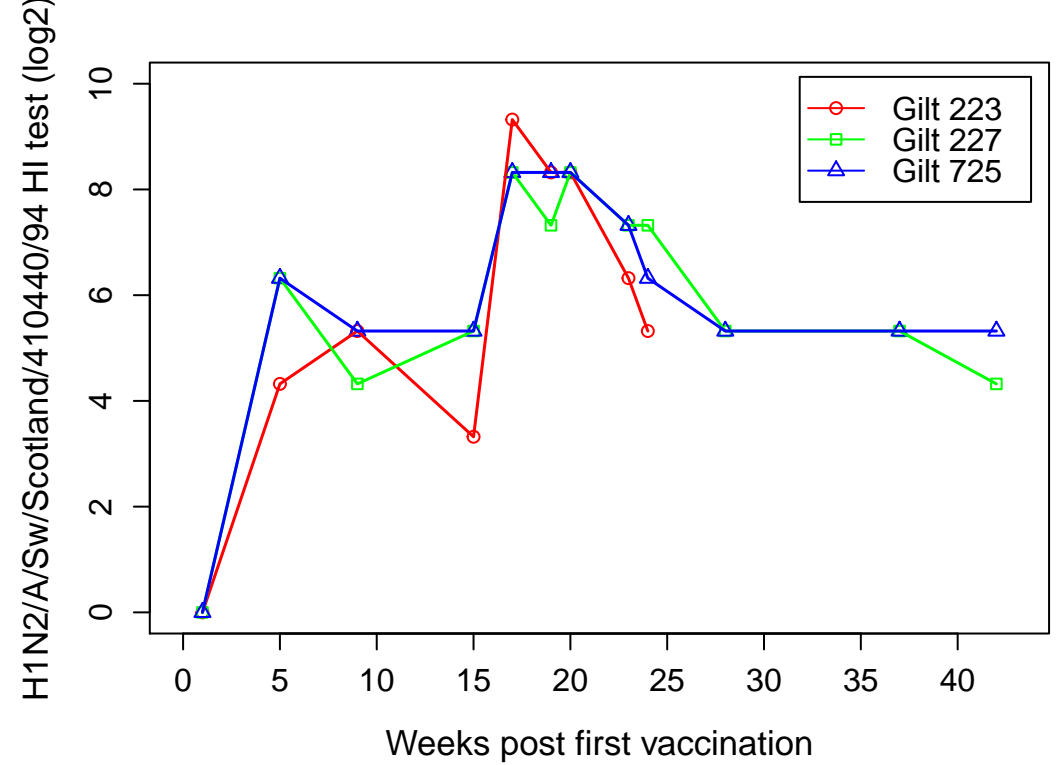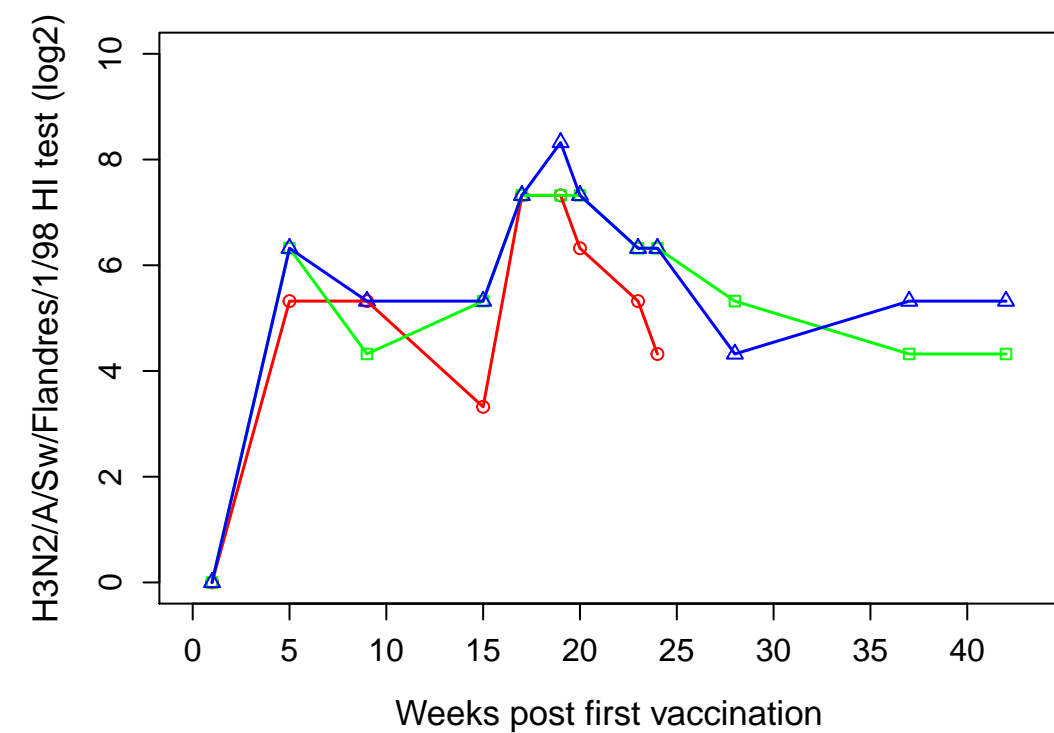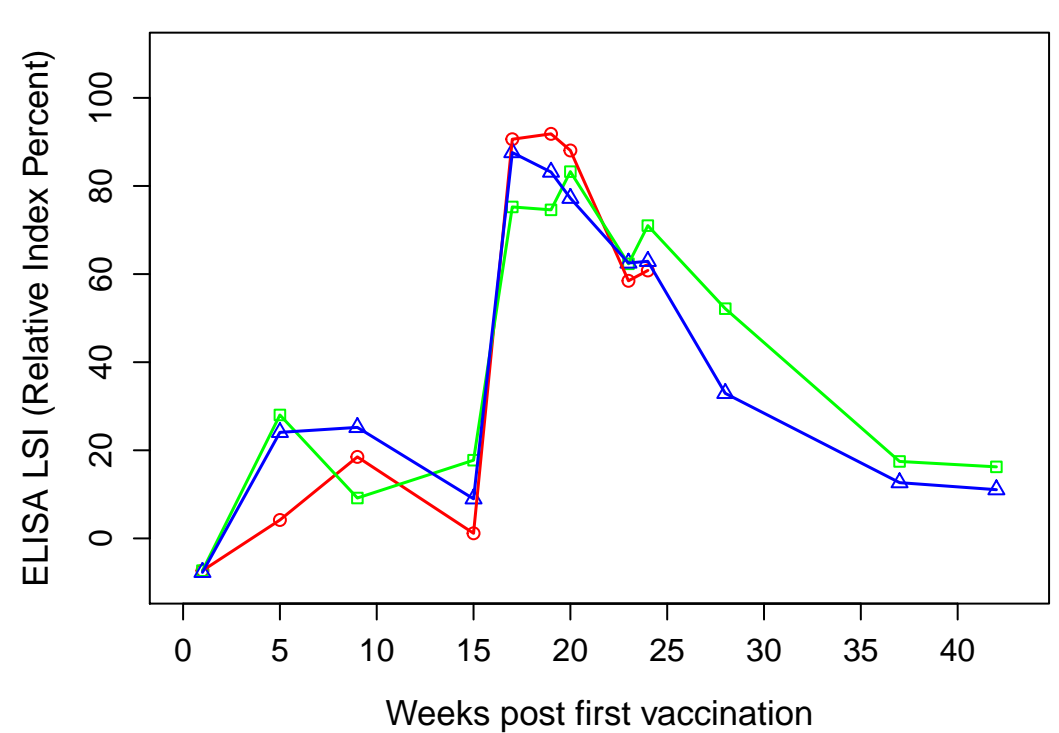

Supplement: Supplementary file 1 — 10.1186/s13567-016-0365-6 Swine influenza A serological profiles of vaccinated gilts. Serological profiles (HI tests and ELISA LSI) of the vaccinated dams starting from first injection (Primo vaccination at weeks 2 and 5, 1st, 2nd and 3rd boosters at weeks 16, 20 and 21 respectively). [file 13567_2016_365_MOESM1_ESM.pdf]
