# Supplementary material for: Maternally-derived antibodies do not prevent transmission of swine influenza A virus between pigs
Source: Vet Res. 2016 Aug 17;47:86. doi: 10.1186/s13567-016-0365-6 (PMC4988049; doi:10.1186/s13567-016-0365-6)
Supplement: Supplementary file 2 — 10.1186/s13567-016-0365-6 Within-host model parameters estimated from individual viral shedding kinetics in contact pigs. The given values correspond to the mean and the 95% confidence interval [95% CI] of estimated individual parameters. [file 13567_2016_365_MOESM2_ESM.docx]

| **Variables/ Parameters** | **Description** | **Units** | **Mean [95% CI]** | **References** |
| --- | --- | --- | --- | --- |
| *T_0_* | Initial number of susceptible target cells | - | 4×10^8^ | [38, 39] |
| *I_0_* | Initial number of infected cells | - | 0 | Assumed |
| *V_0_* | Initial number of free virions | viral copies/10^4^ copies of $\beta$-actin | 0.1 | Assumed, based on [38, 40] |
| *α* | Infection rate of susceptible target cells | (viral copies/10^4^ copies of $\beta$-actin)^-1^.day^-1^ | 1.5×10^-3^ [1.9×10^-5^ - 4.1×10^-3^] | Fitted |
| *δ* | Clearance rate of infected cells | day^-1^ | 2.3 [1.2 - 3.8] | Fitted |
| *p* | Production rates of free virions | (viral copies/10^4^ copies of $\beta$-actin) .day^-1^ | 4.6×10^-3^ [8.5×10^-5^ – 2.7×10^-2^] | Fitted |
| *c* | Virus clearance rate | day^-1^ | 2.5 [1.3 - 3.9] | Fitted |
